# The role of HDAC11 in age-related hearing loss: Mechanisms and therapeutic implications

**DOI:** 10.1515/biol-2025-1086

**Published:** 2025-07-08

**Authors:** Lina Guan, Jing Chen, Hongqun Jiang

**Affiliations:** Department of Otorhinolaryngology Head and Neck Surgery, The First Affiliated Hospital of Nanchang University, 17 Yongwai Zhengjie, Donghu District, Nanchang City, Jiangxi Province, 330000, China

**Keywords:** age-related hearing loss, HDAC11, mitophagy, Pink1/Parkin, Pink1/Parkin pathway, apoptosis and senescence

## Abstract

This study focuses on the critical role of HDAC11 in age-related hearing loss and its underlying mechanisms. Through cellular experiments, we deeply explored the effects of HDAC11 on the proliferation and senescence of HEI-OC1 cells. The results showed that HDAC11 overexpression significantly reduced the acetylation level of α-microtubule protein, which in turn affected the stability of microtubule structure and accelerated the apoptosis and senescence process of HEI-OC1 cells. In addition, the overexpression of HDAC11 inhibited the Pink1/Parkin signaling pathway, which impeded the mitochondrial autophagy process and ultimately led to mitochondrial dysfunction. In animal experiments, we further verified the ameliorative effect of HDAC11 overexpression on hearing loss in aged mice. The experimental results showed that HDAC11 overexpression not only attenuated the histopathological damage of the cochlea in aged mice but also effectively improved their hearing function. Notably, HDAC11 overexpression suppressed the expression of cellular autophagy-related proteins and Pink1 and Parkin proteins. In summary, the present study preliminarily revealed that HDAC11 may regulate mitochondrial autophagy by inhibiting the Pink1/Parkin pathway, thus providing a new theoretical basis for improving hearing loss in the elderly.

## 1 Introduction

Age-related hearing loss (ARHL), also known as presbycusis, is a progressive decline in auditory function that occurs with advancing age. This form of hearing impairment predominantly affects high-frequency sounds and tends to worsen over time [1,2]. ARHL arises from the interplay of multiple factors, including genetic predisposition, environmental influences, metabolic processes, and physiological changes [1–3]. The hair cells located within the inner ear serve as sensory receptors that transduce sound waves into neural signals for transmission to the brain for auditory perception. These cells are categorized into two types: inner hair cells (IHCs) and outer hair cells (OHCs) [4]. OHCs play a crucial role in sound amplification and enhancement of auditory sensitivity, particularly for high-frequency stimuli. Aging is associated with a decline in the number and functionality of OHCs, which constitutes one of the primary mechanisms underlying ARHL [5]. This cellular degeneration results in diminished cochlear sensitivity to high-frequency sounds, leading to significant high-frequency hearing loss. IHCs facilitate the transmission of auditory signals to the auditory nerve; however, while IHC loss also occurs with age, its impact on ARHL is generally less pronounced than that associated with OHC loss [6]. Consequently, investigating the mechanisms governing OHC depletion and functional decline in ARHL is critical for developing effective therapeutic strategies.

Microtubules, a heterodimer composed of alpha- and beta-tubulin proteins play critical roles in various cellular functions, including intracellular transport, maintenance of cell morphology, and establishment of cell polarity [7]. In the mammalian cochlea, extensive expression of acetylated tubulin at different developmental stages suggests a significant relationship between its expression and cochlear development [8]. Histone deacetylase 11 (HDAC11), a deacetylase enzyme, has been found to be notably upregulated in both IHCs and OHCs of aged mice. This upregulation of HDAC11 has been identified as the only deacetylase exhibiting such upregulation within these cells according to experimental data. It has been demonstrated that HDAC11 can reduce levels of alpha-tubulin acetylation [9]. Consequently, we hypothesize that elevated levels of HDAC11 protein in the IHCs of aged mice lead to decreased alpha-tubulin acetylation levels, resulting in diminished microtubule stability.

The Pink1/Parkin pathway plays a pivotal role in mitophagy within cells, primarily responsible for the identification and elimination of damaged mitochondria [10]. In healthy mitochondria, PTEN-induced kinase 1 (Pink1) is translocated to the inner mitochondrial membrane, where it undergoes rapid degradation. However, when mitochondria are compromised and the membrane potential diminishes, Pink1 cannot be effectively translocated, resulting in its accumulation on the outer mitochondrial membrane. This accumulated Pink1 undergoes phosphorylation at the outer mitochondrial membrane and subsequently recruits and phosphorylates E3 ubiquitin ligase Parkin [11]. With advancing age, expression levels of PINK1, Parkin, and BNIP3 in both the auditory cortex and hypothalamus of mice exhibit a significant decline [12]. These findings suggest that dysfunction in age-related mitochondrial autophagy may precipitate cellular alterations within the central auditory system of older individuals, thereby contributing to ARHL. Consequently, inducing mitochondrial autophagy presents a promising strategy for addressing ARHL.

This study investigates the relationship between mitochondrial autophagy, which is mediated by the acetyltransferase HDAC11 protein, and the proliferation of HEI-OC1 hair cells in the mouse cochlea. The findings indicate that the expression of HDAC11 promotes apoptosis and senescence in HEI-OC1 cells, resulting in a reduction of α-tubulin acetylation levels and decreased microtubule stability. Furthermore, the results demonstrate that HDAC11 overexpression leads to a significant decline in mitochondrial membrane potential within HEI-OC1 cells and that this is associated with inhibition of the Pink1/Parkin pathway and suppression of mitochondrial autophagy and ultimately contributes to mitochondrial dysfunction.

## 2 Materials and methods

### 2.1 Cell culture and cell transfection

HEI-OC1 hair cells derived from the mouse cochlea were obtained from Guangzhou Yuanjin Biotechnology Co., Ltd., and cultured in high-glucose Dulbecco’s Modified Eagle’s Medium (DMEM; Gibco, USA) supplemented with 10% fetal bovine serum (Gibco) and 0.06% w/v penicillin at 33°C in a humidified incubator with 5% CO_2_ and saturated humidity. The pcDNA3.1 empty vector and the pcDNA3.1-HDAC11 vector were supplied by Suzhou ZKZH Biotechnology Co., Ltd. (Suzhou, Chian), facilitating the expression of HDAC11 in HEI-OC1 cells. Cell transfection was conducted using Lipofectamine 3000 transfection reagent following the manufacturer’s instructions.

### 2.2 Cell apoptosis was detected by flow cytometry

Following transfection, 1 × 10^6^ cells were collected and washed with phosphate-buffered solution (PBS) by centrifugation at 1,500 rpm for two cycles of 3 min each. The cells were then resuspended in 300 µl of precooled 1× Annexin V-FITC binding solution, to which 5 µl of Annexin V-FITC and 10 µl of PI were added per well. The mixture was gently vortexed, incubated in the dark at room temperature for 10 min, and subsequently analyzed using a flow cytometer (NovoCyte 2060R, Aishen Hangzhou, China) [13].

### 2.3 β-Galactosidase staining

The aging β-galactosidase staining kit (Beyotime, Shanghai, China) was utilized in accordance with the manufacturer’s instructions. Briefly, cells were plated onto six-well plates and subjected to pretreatment. Following gentle washing with PBS, the cells were fixed using a fixative and subsequently washed three times with PBS. Thereafter, 1 ml of staining solution was added to each well and sealed with parafilm; the cells were then incubated at 37°C without CO_2_ overnight.

### 2.4 Measurement of mitochondrial transmembrane potential

The mitochondrial transmembrane potential (ΔΨm) was estimated by monitoring the fluorescent aggregation of JC-1 (Solaybio, Beijing). In brief, HEI-OC1 cells were seeded at a density of 2 × 10^5^ cells per well in six-well plates and subjected to specified conditions. Following washing with pre-warmed serum-free DMEM, the cells were incubated with 2.5 μg/ml JC-1 at 37°C for 30 min and subsequently analyzed using flow cytometry.

### 2.5 Mitochondrial fluorescent probe staining analysis

Mitochondrial staining was conducted using the mitochondrial probe MitoTracker Red CMXRos (Yeasen, Shanghai, China) in accordance with the manufacturer’s protocol. Following PBS washes, cells were counterstained with 4′,6-diamidino-2-phenylindole for 10 min, and images were acquired using an Olympus BX63 microscope (Olympus, Japan).

### 2.6 Immunofluorescence

Each group of cells was fixed with 4% paraformaldehyde at −20°C for 15 min. Subsequently, the cells were washed three times with 1× PBS (5 min per wash). Immunostaining was then conducted. The cells were blocked using 1% Triton X-100 and 5% bovine serum albumin (BSA) in PBS for 1 h.

### 2.7 Immunofluorescence

Each group of cells was fixed with 4% paraformaldehyde at −20°C for 15 min. Subsequently, the cells were washed three times with 1× PBS (5 min per wash). Immunostaining was then conducted. The cells were blocked using 1% Triton X-100 and 5% BSA in PBS for one hour.

### 2.8 Animal grouping and interventions

C57BL/6 mice (12-month-old, male, body weight 35 ± 3 g) and C57BL/6 mice (7-week-old, male, body weight 20 ± 2 g) were purchased from Henan Scripps Bio-technology Co., Ltd. (license: SCXK (Yu) 2020-0005, Zhengzhou, China). In the SPF animal laboratory, all mice were raised at 22–24°C, maintained at a humidity range of 60–75%, and followed a 12-h daytime to 12-h nighttime cycle.

Male C57/BL mice (SPF grade) were randomly divided into four groups: the young group (7 weeks old), the old group (12 months old), the old + NC group, and the old + HDAC11 group, totaling four groups. The old + NC group and the old + HDAC11 group were injected by tail vein with airborne adenovirus and HDAC11 overexpression adenovirus (both airborne adenovirus and HDAC11 overexpression adenovirus were provided by Suzhou ZKZH Biological Company), and the young and old groups were injected with equal amounts of saline.


**Ethical approval:** The research related to animal use has been complied with all the relevant national regulations and institutional policies for the care and use of animals and has been approved by the Animal Care and Use Committee of the First Affiliated Hospital of Nanchang University.

### 2.9 Auditory brainstem response (ABR)

The ABR of each group of mice was measured within 24 h after the completion of the intervention. First, the mice were weighed, anesthetized by intraperitoneal injection of 1.2% tribromoethanol solution, and then placed on a heating pad in a soundproof room at a temperature of about 37°C. After the anesthesia was well established, the recording electrodes were placed subcutaneously at the midpoint of the line connecting the two ear margins of the mice, and the reference electrode was placed subcutaneously in the measurement ear, with the ground wire connected subcutaneously in the contralateral ear. For the experimental stimulus sounds, we used short, acoustic (Click) as well as 4, 8, 16, 24, and 32 kHz short pure tones (Tone burst, rise/fall time 0.5 ms). We used Tucker Davis Technology (TDT, Alachua, USA) and BioSigRZ software for ABR testing. The number of superimpositions was 521. The stimulus sound intensity started at 90 dB SPL and was gradually decreased in 10 dB steps until no waveform was detected to determine the threshold.

### 2.10 Hematoxylin-eosin (HE) staining

The mice were executed after the last ABR test, and the cochlear tissues were extracted and immersed in 4% paraformaldehyde fixative (the volume ratio of the tissue to the fixative was about 1:10), fixed at 4°C for 24 h, then rinsed with PBS, and decalcified in EDTA decalcification solution for 3 days, and the decalcification was completed for paraffin sectioning. After paraffin sections were baked, dewaxed and hydrated, stained with hematoxylin stain for 3–5 min, rinsed with running water, differentiated with 1% hydrochloric acid alcohol, counterblue with counterblue solution, and stained with eosin for 3–5 min, the sections were dehydrated, sealed, and observed under a microscope (BX43, Olympus).

### 2.11 Reactive oxygen species (ROS), malondialdehyde (MDA), and superoxide dismutase (SOD) measurement

ROS, MDA, and SOD levels in the cochlear tissues of each group of mice were detected using ROS Assay Kit (Elabsciense, China), MDA Assay Kit (Elabsciense, China), and SOD Assay Kit (Elabsciense, China). The assays were performed according to the steps of the manufacturer’s instructions.

### 2.12 Western blot (WB)

Proteins were extracted from cells or cochlear tissue using RIPA buffer (Thermo Plus, USA). A total of 20 μg of protein samples was electrophoretically separated on a 10% SDS polyacrylamide gel and subsequently transferred to a polyvinylidene fluoride membrane (Millipore, Burlington, MA, USA). Following blocking with 5% skim milk, the membrane was incubated overnight at 4°C with anti-HDAC11 (1:3,000, Affinity, China), anti-LC3 II/I (1:3,000, Affinity, China), anti-p62 (1:1,000, Affinity, China), anti-Beclin-1 (1:1,000, Affinity, China), anti-Atg5 (1:1,000, Affinity, China), anti-p-AMPK (1:1,000, Affinity, China), anti-Pink1 (1:3,000, Affinity, China), and anti-Parkin (1:3,000, Affinity, China). The membrane was then incubated with secondary antibodies (1:3,000) at room temperature for 1 h. Immunoreactive bands were detected using enhanced chemiluminescence (Millipore, USA) and analyzed for gray values using ImageJ software (NIH, USA). β-Actin served as the internal reference protein [14].

### 2.13 Statistical analysis

All results were expressed as mean ± standard deviation. Statistical analysis was performed using GraphPad Prism Software 9 (GraphPad Software, USA). The comparisons between groups were conducted using the one-way analysis of variance with Tukey’s *post hoc* test. The *p*-value of <0.05 was considered statistically significant.

## 3 Results

### 3.1 Overexpression of HDAC11 promotes apoptosis and cellular senescence in HEI-OC1 cells

**Figure 1:**
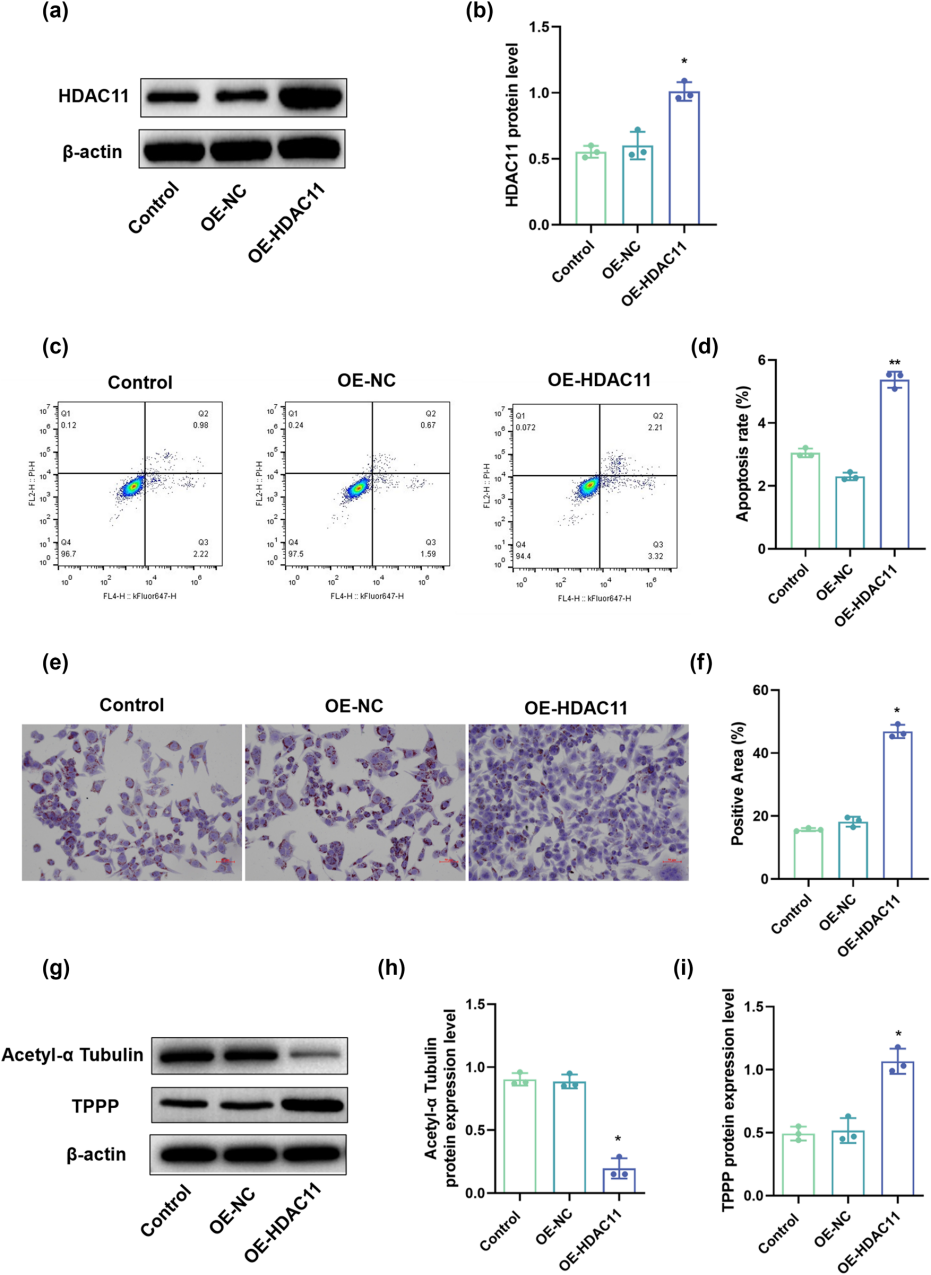
Effect of HDAC11 on apoptosis, senescence, and microtubule stability in HEI-OC1 cells. (a) Representative WB detection strip. (b) HDAC11 protein expression level. (c and d) Flow cytometry detection of apoptosis. (e and f) β-Galactosidase staining to observe cell senescence in each group. (g) Representative WB detection strip. (h and i) Acetyl-α Tubulin and TPPP protein expression level. *P < 0.05 vs control.

Initially, the HDAC11 overexpression vectors were transfected into HEI-OC1 cells, with transfection efficiency verified by Western blot analysis. The results indicated (Figure 1a–c) that, compared to the control group, the expression level of HDAC11 protein in the OE-HDAC11 group was significantly elevated (*P* < 0.05), confirming successful transfection. Apoptosis was assessed using flow cytometry, revealing that (Figure 1d and e): relative to the control group, apoptosis rates in the OE-HDAC11 groups were significantly higher (*P* < 0.05). Cellular senescence was evaluated through β-galactosidase staining; results demonstrated (Figure 1f and g) that compared to the control group, there was a marked increase in senescence ratios within OE-HDAC11 groups (*P* < 0.05). Collectively, these findings suggest that overexpression of HDAC11 promotes apoptosis and cellular aging in HEI-OC1 cells.

### 3.2 Effect of HDAC11 overexpression on microtubule stability in HEI-OC1 cells

To further elucidate the effects of HDAC11 overexpression on microtubule stability in HEI-OC1 cells, we assessed the acetylation levels of α-tubulin protein and the expression of tubulin polymerization-promoting protein TPPP via Western blotting. The results indicated (Figure 3a–c) that, compared to the control group, acetyl-α tubulin protein levels in the OE-HDAC11 group were significantly reduced (*P* < 0.05), while TPPP protein expression was significantly elevated. This suggests that HDAC11 overexpression can diminish α-tubulin acetylation and enhance microtubule polymerization.

### 3.3 Overexpression of HDAC11 leads to mitochondrial dysfunction in HEI-OC1 cells

**Figure 2:**
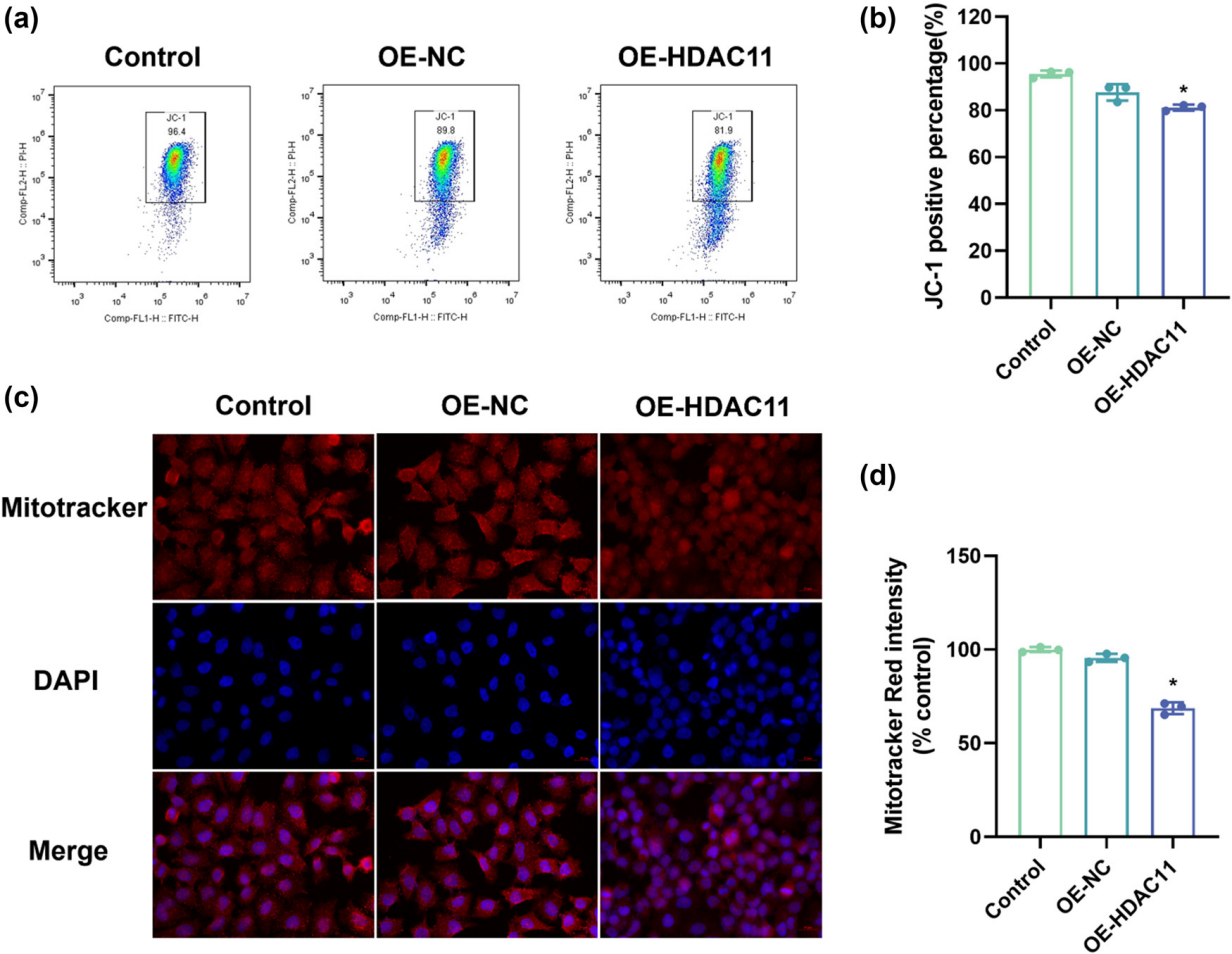
Mitochondrial dysfunction in HEI-OC1 cells caused by overexpression of HDAC11. (a and b) Changes in mitochondrial membrane potential detected by flow cytometry. (c and d) Changes in mitochondrial membrane potential detected by Mitotracker Red fluorescent staining. *P < 0.05 vs control.

To assess the impact of HDAC11 overexpression on mitochondrial function in HEI-OC1 cells, alterations in mitochondrial membrane potential were evaluated using flow cytometry and Mitotracker Red fluorescence staining. The flow cytometry results indicated (Figure 2a and b) that the mitochondrial membrane potential in both the OE-HDAC11 groups was significantly reduced compared to the Control group (*P* < 0.05). Similarly, Mitotracker Red fluorescence staining results (Figure 2c and d) demonstrated that the mitochondrial membrane potential in the OE-HDAC11 groups was markedly lower than that of the control group (*P* < 0.05). These findings suggest that overexpression of HDAC11 may contribute to mitochondrial dysfunction in HEI-OC1 cells.

### 3.4 Overexpression of HDAC11 inhibited mitophagy in HEI-OC1 cells

**Figure 3:**
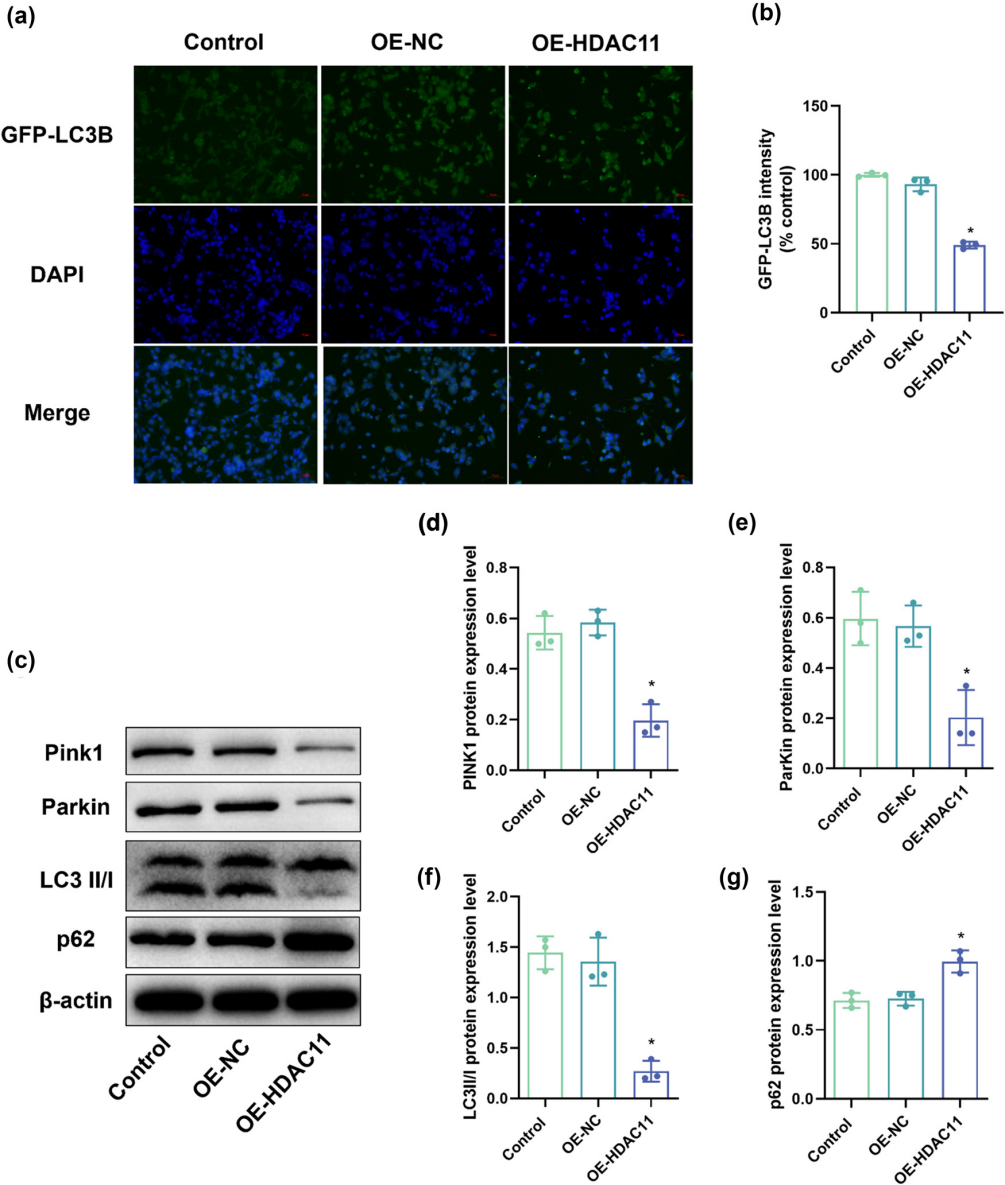
HDAC11 overexpression inhibits mitophagy in HEI-OC1 cells. (a and b) Immunofluorescence observation of cell LC3B protein expression. (c) Representative WB detection strip. (d–g) Mitophagy-related proteins Pink1, Parkin, LC3 II/I, and p62 protein expression. *P < 0.05 vs control.

**Figure 4:**
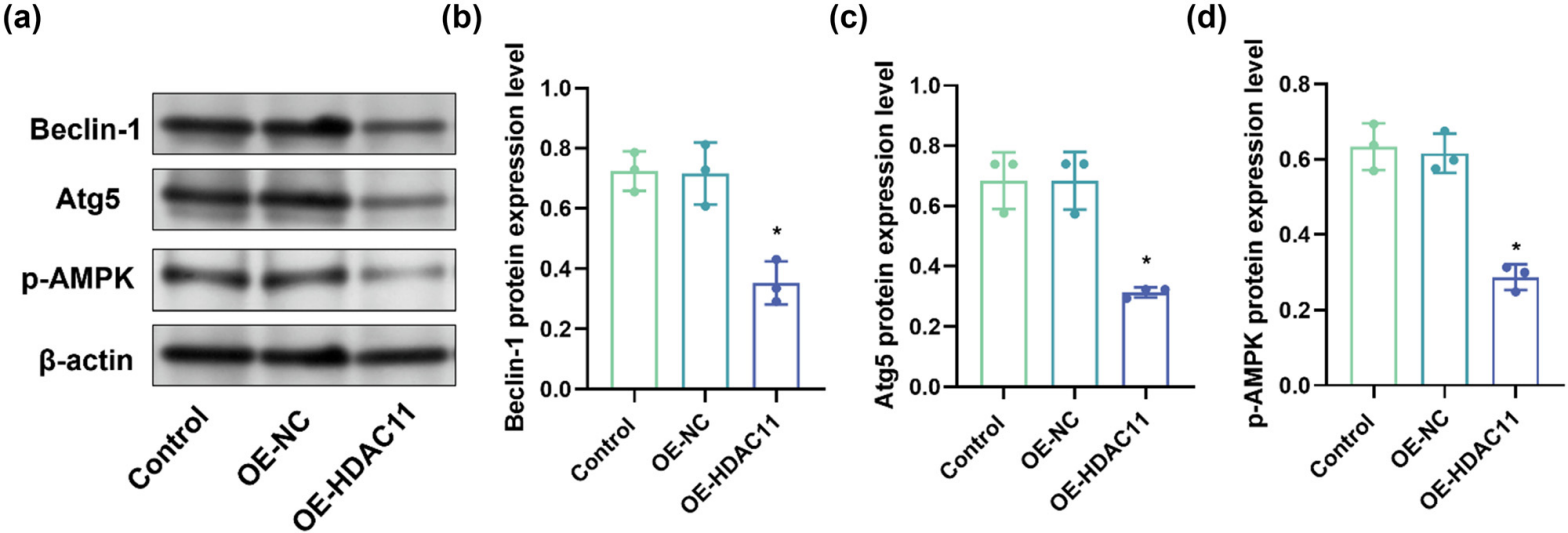
HDAC11 overexpression decreases Beclin-1, Atg5, and p-AMPK protein levels in HEI-OC1 cells. (a) Representative WB detection strip. (b–d) Beclin-1, Atg5, and p-AMPK protein expression. *P < 0.05 vs control.

To further evaluate the effect of HDAC11 overexpression on mitophagy in HEI-OC1 cells, we detected the expression of LC3B using immunofluorescence and analyzed the expression of autophagy-related proteins microtubule-associated protein 1 light chain 3 (LC3) II/I, ATP-dependent RNA helicase p62 (p62), Beclin-1, Atg5, and the level of phosphorylation of 5′-AMP-activated protein kinase subunit beta-1 (AMPK) protein by Western blotting. Immunofluorescence results (Figure 3a and b) showed that the proportion of LC3B-positive cells was all significantly lower in the OE-HDAC11 group compared with the control group (*P* < 0.05). Western blot analysis showed that compared with the control group, the expression of LC3 II/I, Beclin-1, Atg5, and p-AMPK was significantly lower in the OE-HDAC11 group (*P* < 0.05), and p62 expression was significantly elevated (*P* < 0.05) (Figures 3c–g and 4). These results suggest that overexpression of HDAC11 may inhibit mitophagy in HEI-OC1 cells.

The Pink1/Parkin pathway plays a key role in mitochondrial autophagy and is mainly responsible for recognizing and removing damaged mitochondria. To further investigate the effect of HDAC11 overexpression on mitochondrial autophagy, we used Western blotting to detect the expression of Pink1 and Parkin proteins in the Pink1/Parkin pathway. The results showed (Figure 3c–e) that the expression of Pink1 and Parkin proteins was significantly lower in the OE-HDAC11 group compared with the control group, while the expression of Pink1 and Parkin proteins was significantly higher in the overexpression group (*P* < 0.05). This suggests that HDAC11 overexpression may inhibit mitochondrial autophagy in HEI-OC1 cells by suppressing the Pink1/Parkin pathway.

### 3.5 HDAC11 overexpression improves hearing loss in ARHL mice

**Figure 5:**
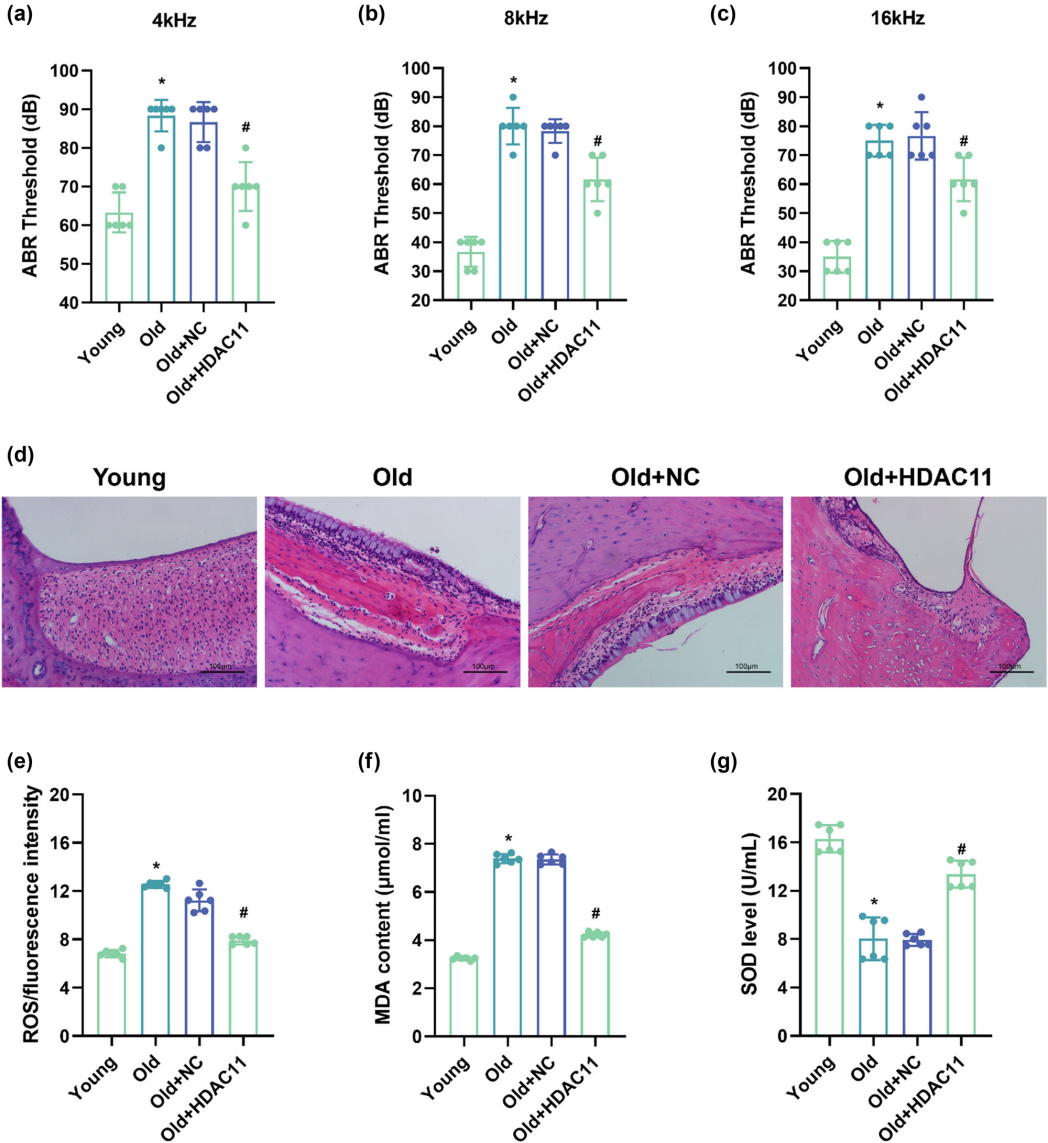
HDAC11 overexpression improves hearing loss in ARHL mice. (a–c) The hearing thresholds of the mice in each group were examined at different frequencies. (d) HE staining was performed on the cochlear tissue. (e–g) ROS, MDA, and SOD levels were examined in the cochlear tissues of mice in each group. *P < 0.05 vs young and ^#^ *P* < 0.05 vs old.

The hearing thresholds of the mice in each group were examined at different frequencies, and the results showed that, compared with the mice in the young group, the mice in the old group exhibited significant hearing impairment at 4, 8, and 16 kHz, with the most pronounced hearing impairment observed at 16 kHz. Compared with the mice in the old group, there was no significant change in the ABR thresholds of the mice in the old + NC group; compared with the mice in the old group, the ABR thresholds of the mice in the old + HDAC11 group were significantly lower (*P* < 0.05), suggesting that the overexpression of HDAC11 has a certain protective effect on the hearing of the old mice (Figure 5a–c). To observe the changes in the internal microstructure of the cochlea, HE staining was performed on the cochlear tissue. Cross-sectional images showed that the cochlear spiral ganglion of young group mice was structurally intact and morphologically well-organized. In contrast, the cochlear tissue of old group mice showed disorganized spiral ganglia and vacuoles. Compared with mice in the old group, mice in the HDAC11 overexpression group showed a significant improvement in cochlear structural arrangement and reduced damage to spiral ganglion neuronal cells (Figure 5d).

To evaluate the effects of HDAC11 on oxidative stress injury in ARHL mice, ROS, MDA, and SOD levels were examined in the cochlear tissues of mice in each group (Figure 5e–g). ROS and MDA levels were significantly higher while SOD levels were significantly lower in the cochlear tissues of mice in the old group compared to the young group. ROS and MDA levels were significantly lower and SOD levels were significantly higher in the cochlear tissues of mice in the old + HDAC11 group compared with the old group.

### 3.6 Effect of HDAC11 overexpression on mitophagy-related protein expression in ARHL mice

**Figure 6:**
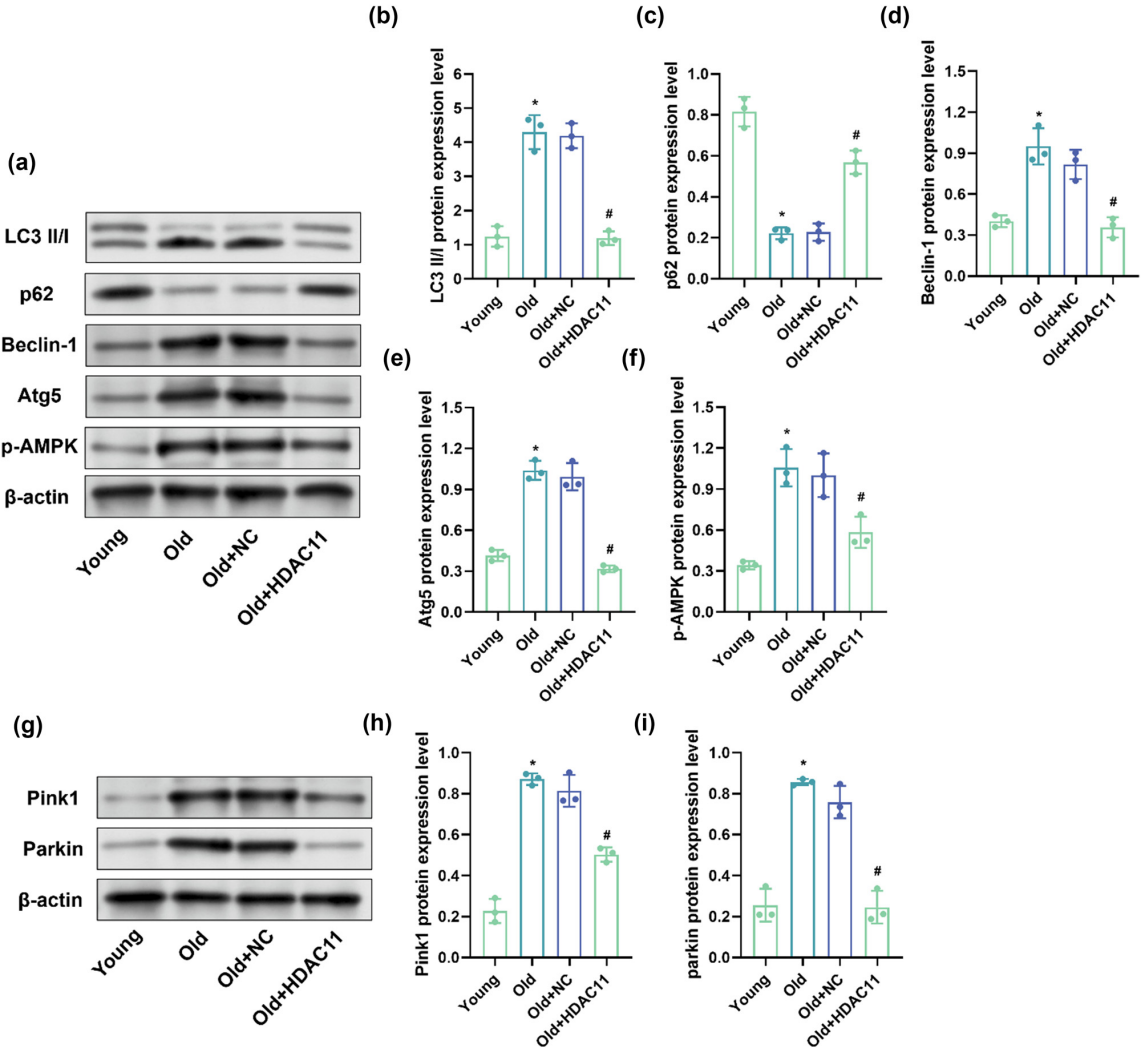
Effect of HDAC11 overexpression on mitophagy-related protein expression in ARHL mice. (a) Representative WB detection strip. (b–f) LC3 II/I, p62, Beclin-1, Atg5, and p-AMPK protein expression level. (g) Representative WB detection strip. (h and i) Pink1 and Parkin protein expression level. *P < 0.05 vs young and ^#^ *P* < 0.05 vs old.

Further, we examined the changes of LC3 II/I, p62, Beclin-1, Atg5, p-AMPK, and Pink1 and Parkin protein expression in cochlear tissues of mice in each group by WB. The protein levels of LC3 II/I, Beclin-1, Atg5, and p-AMPK were significantly higher (*P* < 0.05), while the protein level of p62 was significantly lower (*P* < 0.05) in the cochlear tissue of mice in the old group compared with mice in the young group; LC3 II/I, Beclin-1, Atg5, and p-AMPK protein levels were significantly lower (*P* < 0.05), while p62 protein levels were significantly higher (*P* < 0.05) in the cochlear tissues of mice in the old + HDAC11 group compared to mice in the old group (Figure 6a–f). The levels of Pink1 and Parkin proteins were significantly higher (*P* < 0.05) in the cochlear tissues of mice in the old group compared to mice in the young group, while the levels of Pink1 and Parkin proteins were significantly lower (*P* < 0.05) in the cochlear tissues of mice in the old + HDAC11 group compared to mice in the old group (Figure 6g–i).

## 4 Discussion

Hearing loss exerts a profound impact on individuals, resulting in communication difficulties, social isolation, and a diminished quality of life. The etiology of hearing loss is multifactorial, encompassing ototoxic drug exposure, noise-induced damage, and age-related auditory decline [15]. The House Ear Institute-Organ of Corti 1 (HEI-OC1) cell line is an auditory cell line derived from the auditory organs of transgenic mice [16]. This study aims to address the role and mechanism of HDAC11 on ARHL. From the cellular level, we explored the effects of HDAC11 on the proliferation and aging of HEI-OC1 cells. The results show that HDAC11 overexpression promotes apoptosis and aging of HEI-OC1 cells by reducing the acetylation level of α-tubulin and affecting microtubules stability. In addition, HDAC11 overexpression inhibits the Pink1/Parkin pathway, thereby inhibiting mitochondrial autophagy in HEI-OC1 cells, resulting in mitochondrial dysfunction. From the animal level, we explored the effect of HDAC11 overexpression on hearing loss in aged mice. Our results show that HDAC11 overexpression can improve hearing loss in older mice and reduce cochlear histopathological damage in older mice. It was also found that HDAC11 overexpression can inhibit the expression of cell autophagy-related proteins and Pink1 and Parkin proteins. Therefore, the results of this study preliminarily believe that HDAC11 may inhibit mitochondrial autophagy by inhibiting the Pink1/Parkin pathway and improve age hearing loss.

Cochlear hair cells rely on a network of internal microtubules to maintain their specific morphology and structure. As part of the cytoskeleton, microtubules not only provide mechanical support to the cell but are also involved in the transport and distribution of intracellular substances [17]. A stable microtubule network ensures that the cilia of hair cells are correctly aligned and maintain their functionality, thus ensuring their sensitivity and responsiveness to acoustic signals. In the face of external stress and damage, a stable microtubule network may help cochlear hair cells repair and protect themselves from damage more effectively. The microtubule system is involved in intracellular transport and cytoskeletal remodeling and is an important component of cellular repair [18]. Therefore, the effect of microtubule stability on cochlear hair cells is not limited to the maintenance of morphology and structure but also involves the overall performance of auditory functions [19]. Our results showed that HDAC11 overexpression caused a decrease in the acetylation level of α-microtubulin and reduced microtubule stability. This suggests that HDAC11 overexpression may affect cochlear hair cell proliferation by decreasing microtubule stability.

Studies have shown that excessive autophagy levels in cochlear cells are a key factor in the development of ARHL. Autophagy is an important mechanism for cells to maintain homeostasis, protecting cells from damage by degrading damaged organelles and proteins. Inner ear hair cells are key cells that sense sound and convert mechanical signals into neural signals, which require very high homeostasis of the intracellular environment. Once the inner ear hair cells are damaged (such as oxidative stress, mechanical damage, etc.), it is necessary to clear the damaged mitochondria and other cellular components through autophagy. Moderate autophagy helps keep cells healthy. When the autophagy process is overactivated, it may instead lead to excessive degradation of important substances in the cell and lead to cell death. In the study of hearing loss, excessive autophagy is believed to be a mechanism that causes hair cell death in the inner ear. Hair cell death can lead to hearing loss because hair cells in the inner ear cannot regenerate, and damage to the inner ear is usually irreversible. Studies have shown that in ARHL, the autophagy process may be dysregulated, leading to further deterioration of cellular function [20]. For example, researchers found that cholesterol metabolism abnormalities and lysosomal dysfunction induce ARHL by inhibiting mTORC1-TFEB-dependent autophagy. By performing experiments in mouse models, the researchers observed increased cholesterol and lipofuscin aggregation in ARHL tissues and that autophagy flow was inhibited by the accumulation of damaged lysosomes and autolysosomes [20]. LC3 is one of the very important proteins in the process of autophagy and it has two main forms: LC3-I and LC3-II. LC3-I is an unmodified form, while LC3-II is a form that binds to the autophagosome membrane after phospholipidation modification, and the accumulation of LC3-II is often regarded as a marker of autophagy activity [21]. Beclin-1 is one of the key regulators of autophagy, which initiates the initial process of autophagy by forming complexes with other proteins (such as Vps34 and Vps15) [22]. Beclin-1 is mainly involved in the formation of autophagosomes and coordinates autophagy activities in cells through the signaling pathway that regulates autophagy [23]. p62 is a multifunctional protein that acts as both a substrate for autophagy and an aptamer during autophagy. p62 can bind to autophagic substrates (such as damaged proteins or organelles) and direct these substrates into autophagosomes through interactions with LC3 [24]. Atg5 is one of the indispensable proteins in the process of autophagy and is involved in the formation of autophagosomes [25]. AMPK is a positive regulator of autophagy and can promote the occurrence of autophagy under energy deficiency or stress conditions, helping cells clear out damaged organelles and waste [26]. In this study, we found that in the cochlear tissues of elderly mice, increased levels of LC3II/I, Beclin-1, Atg5, and p-AMPK proteins and decreased levels of p62 proteins were observed. After HDAC11 overexpression, the expression level of these proteins in the cochlear tissue of elderly mice was reversed. This suggests that the effect of HDAC11 overexpression to improve hearing loss may be associated with inhibition of cell autophagy.

In recent years, increasing studies have revealed the close relationship between mitochondrial dynamics and hearing loss. The role of mitochondria in the auditory system is mainly reflected in maintaining the energy supply of inner ear cells, regulating the concentration of calcium ions in the cells, and removing damaged oxidized substances [27]. Hair cells and auditory nerve cells in the inner ear are very sensitive to oxidative stress, and mitochondrial dysfunction can lead to cell death and ultimately hearing loss [28]. As you age or due to oxidative stress, damaged mitochondria need to be effectively removed, otherwise, waste that damages cellular function will accumulate. Mitophagy plays an important role in the physiological function of inner ear cells [29]. Studies have shown that mitochondrial dynamic mechanisms related to hearing loss include Pink1/Parkin signaling pathway, oxidative stress, and mitochondrial dysfunction [30]. Pink1 is a mitochondrial membrane protein that senses mitochondrial damage and activates Parkin (an E3 ubiquitin ligase), thereby starting the mitochondrial removal process [31]. In a variety of hearing loss models, dysfunction of the Pink1/Parkin signaling pathway is closely related to hearing loss. For example, the mutated Pink1 gene or Parkin gene exhibits early hearing loss in animal models. Inner ear cells are particularly susceptible to oxidative stress, which may impair mitochondria function, leading to loss of mitochondrial membrane potential, reduced ATP synthesis, and accumulation of calcium ions in mitochondria [32]. Prolonged oxidative stress may lead to damage to hair cells by changing the dynamics of mitochondria (such as inhibiting mitochondria division or fusion process) [33]. Our results show that in cell experiments, HDAC11 overexpression can reduce HEI-OC1 mitochondrial membrane potential and reduce cell Pink1 and Parkin protein levels; in animal experiments, HDAC11 overexpression can improve hearing loss in elderly mice and improve aging. Oxidative stress damage in mice inhibits the levels of Pink1 and Parkin protein in cochlea in elderly mice. These results suggest that HDAC11 may improve age hearing loss by inhibiting mitophagy by inhibiting the Pink1/Parkin signaling pathway.

In summary, the results of this study preliminarily suggest that HDAC11 may inhibit mitochondrial autophagy by inhibiting Pink1/Parkin pathway and ameliorating ARHL. However, there are some limitations in this paper. In this article, the role of HDAC11 in ARHL was explored only at the cellular and animal levels, and its clinical role remains unclear. This study confirmed that HDAC11 inhibits mitochondrial autophagy defects in ARHL by inhibiting the Pink1/Parkin pathway, and targeting HDAC11 can significantly improve hearing function in elderly mice. Although the challenges of inhibitor selectivity and delivery efficiency still need to be overcome for clinical translation, the unique mechanism of action of HDAC11 may make it a potential broad-spectrum target for ARHL and other age-related diseases. Future studies could explore combination therapies with HDAC11 inhibitors and mitochondrial protectors, or develop cochlea-targeted nanodelivery systems to drive the leap from basic discovery to clinical application.

## Supplementary Material

Supplementary Figure
